# Assessment of general population knowledge, attitude, and practice on safe unused and expired drugs disposal: a cross-sectional study

**DOI:** 10.12688/f1000research.142146.1

**Published:** 2023-10-16

**Authors:** Abd-ul Munaf Mohammed, Fadya Al-Hamadani

**Affiliations:** 1 Clinical Pharmacy, College of Pharmacy, University of Baghdad, Baghdad, 10045, Iraq

**Keywords:** Attitude, Drugs Disposal, Knowledge, Practice, Unused medicines.

## Abstract

**Background:**

The appropriate disposal of medication is a well-recognized issue that has convened growing recognition in several contexts. Insufficient awareness relating to appropriate methods for the disposal of unneeded medicine may result in notable consequences. The current research was conducted among the public in Iraq with the aim of examining their knowledge, attitude, and practices regarding the proper disposal of unused and expired medicines.

**Methods:**

The present study used an observational cross-sectional design that was community-based. The data were obtained from using an online questionnaire. The study sample included people of diverse genders, regardless of their race or occupational status. The study mandated that all participants meet two criteria: firstly, they had to be a minimum of 18 years old, and secondly, they needed to have permanent residency status in Iraq.

**Results:**

The research population consisted of 591 participants from general population. The majority of participants were men (64.8%) with average age of 33.5±9.1 years. The majority of participants (˃50%) answered four out of eight knowledge questions correctly. There were significant (P-value ˂0.05) differences in four knowledge items about disposing of unused/expired medications according to the educational level. Elder participants had significantly higher knowledge about the disposing of unused/expired medications compared to younger ones. More than 84% correctly agreed that improper disposal of unused and expired medication has adverse effects on environment. Although the majority of the participants had positive attitude toward the safe disposal of unused/expired medications, their actual practice was improper for these medications. Approximately 70% of the participants have disposed unused/expired medications into their home trash can.

**Conclusion:**

Despite increasing awareness from the general populace about the possible harm and substantial hazards linked to expired medicines, there is an absence of understanding concerning the proper techniques for disposing of and managing these prescriptions.

## Introduction

The proper disposal of medicine is a well-acknowledged challenge that has gained increasing attention in diverse settings. ^1^^,^^2^ A lack of sufficient knowledge regarding the proper methods of disposing of unused medication can lead to significant consequences, such as the accumulation of chemical compounds and dangerous chemicals from the medication in the environment around it, unintentional events of overdose, and the possibility of prescription drug misuse. ^3^^,^^4^ A number of countries have implemented legislation pertaining to the appropriate disposal of unwanted or expired medicine. ^5^ Ensuring the effectiveness and safety of pharmaceutical items may be achieved by following the prescribed storage conditions specified on the label and using them prior to their designated expiry date. ^6^^,^^7^ The disposal of pharmaceuticals is often advised by suppliers and certain healthcare organizations, taking into consideration their expiry date. ^8^^,^^9^ This is primarily because outdated medicines could decompose, resulting in a loss of effectiveness or even the manifestation of harmful consequences. ^10^ Prescribed drugs often remain unused owing to several circumstances, including changes in treatment plans, unpleasant responses, improvements in patients’ well-being, or other contributing factors. ^11^^–^^14^ According to the World Health Organization (WHO), a considerable number of pharmaceuticals has been prescribed or made available for sale improperly, resulting in the buildup of enormous volumes of solid waste consisting of expired and unused medications, hence, there is a notable burden for the environment that is linked to the disposal of these pharmaceutical substances. ^15^

The Food and Drug Administration (FDA) in the United States has issued guidelines to the general public pertaining to the proper disposal of leftover pharmaceuticals; as per the guidelines provided by the FDA, the optimal approach for disposing of unneeded or expired medications involves depositing them at certified drug take-back locations, these locations are often available at various medical institutions, including retail pharmacies and health centers. ^16^ Properly disposing of expired, unwanted, or unused drugs by customers is a significant worldwide concern. ^17^ Numerous developed countries have implemented initiatives to recycle unwanted medications properly. In Australia and Canada, the National Return and Disposal of Unwanted Medicines Project has been implemented with the respective governments and pharmaceutical industries. ^18^ Drug take-back strategies are prevalent in both the United Kingdom and Sweden. ^19^

A study conducted in Africa examined the knowledge and perceptions of people regarding the disposal procedures for unused medications. The findings revealed that families lacked knowledge regarding proper disposal procedures. This poor knowledge can be attributed to insufficient public education and promotion campaigns and the failure of healthcare providers to deliver disposal advice at healthcare facilities and drug stores. Additionally, unclear disposal instructions on medicine packages and a disregard for reading these instructions were identified as contributing factors to this issue. ^20^ Despite the existence of international guidelines regarding the appropriate disposal practices for expired and unused drugs, ^21^ implementation of these guidelines is often inadequate, and the public has limited access to sometimes conflicting information on this matter. ^22^^,^^23^

Furthermore, it is important to acknowledge that certain medications may be appropriately discarded by flushing them down the toilet, as long as they are included in FDA roster of pharmaceuticals that are deemed safe for such disposal. The list provided includes various opiates such as buprenorphine, morphine, other derivatives of opioids, diazepam, and methylphenidate. ^24^ In contrast, medicines that are not acceptable for disposal by flushing should be mixed with a less appealing substance, such as dirt, cat litter, or recently used coffee grounds; following this, it is recommended that they be securely sealed inside a hermetically sealed plastic bag and appropriately discarded in the designated waste receptacle. ^25^

The existing body of research on the knowledge and attitudes of the general population regarding the disposal of expired and unused medicines has been conducted in various regions worldwide. However, the specific knowledge and attitudes of the Iraqi population regarding the safe removal of expired and unused drugs remain unknown. This lack of understanding is primarily due to the absence of a regulatory authority system in Iraq that addresses the proper handling of expired and unused medicines at the household level. Therefore, this study aimed to evaluate the general population’s knowledge, attitude, and practices about the disposal procedures for expired and unused medications.

## Methods

### Ethics statements

The research proposal obtained approval from the Central Scientific Committee of the College of Pharmacy at the University of Baghdad (approval number: RECAUBCP292023A on 29/1/2023). The ethical committee waived the requirement for obtaining written informed consent, as all participants voluntarily participated in the study. All the procedures were conducted in adherence to the appropriate guidelines and regulations.

### Study design

This research utilized an observational cross-sectional design, community based, and was conducted from the 28th of Feb to the 11th of May 2023. The data were collected by an online questionnaire that was administered on a voluntary basis without offering any incentives to motivate participation. The researchers provided a detailed explanation of the study’s scope and objective in the introductory section of the questionnaire. Privacy and confidentiality were secured through the study items.

### Sample size

The estimation of the sample’s size was conducted with the Raosoft ^®^ online software calculator. The estimate was determined based on the need to achieve a minimum sample size, set a confidence level of 95%, and maintain a margin of error of 5%. A recommendation was made to use a sample size of 591 individuals. The online questionnaire portal was closed after it reached the above specified number.

### Inclusion criteria

Adults who agree to participate, and are at least eighteen years old.

### Exclusion criteria

Any healthcare workers, and participants under the age of eighteen.

### Questionnaire

The questionnaire included 23 questions and was divided into four distinct sections. The first section of the study collected socio-demographic data, such as age, gender, educational attainment, marital status, monthly income, and sources of information on drug disposal. The three remaining components consisted of a series of conceptual inquiries that assessed people’ knowledge (8 questions), attitudes (10 questions), and practices (5 questions) pertaining to the disposal of drugs. This study thoroughly analyzes the existing literature to identify relevant inquiries about knowledge, attitudes, and practices (KAP) in the context of unused drugs management. These questions were adopted from various studies ^7^^,^^13^^,^^26^^–^^31^; furthermore, it was later modified to conform to the precise aims of the present study. We collected the accurate responses, awarding one point for each right answer, with the exception that selecting the “I do not know” option was considered an incorrect answer. The attitude score was measured using a 5-point Likert scale.

The content validity of each survey question was assessed by a panel of five experts who are affiliated with the Central Scientific Committee of the clinical department at the College of Pharmacy, University of Baghdad. An agreement was established by the experts about the importance and clarity of each item included in the question. Subsequently, the study proceeded to a preliminary phase, with a cohort of 48 individuals, with the aim of assessing its clarity and reliability. The reliability of the questionnaire was assessed by calculating the alpha-Cronbach’s coefficient, which yielded a value of 0.67.

### Data collection

An online questionnaire was developed using the Google Forms platform in order to assess the knowledge, attitudes, and practice of the general population about the disposal of drugs. Following this, the survey was distributed across several social media sites (Facebook, WhatsApp, Telegram). The data were collected through a convenient method of sampling. The participants were formally notified that their participation in the research endeavor was totally voluntary and that their replies would be handled with the utmost regard for anonymity and confidentiality. The survey included a combination of closed-ended and open-ended inquiries, with the objective of collecting information on the socio-demographic attributes, knowledge, attitudes, and behaviors of the participants. The completion of the questions normally necessitates a time range of probably 10 to 15 minutes.

### Bias

In this type of research, sometimes certain individuals may have refrained from disclosing their improper medicine disposal practices in order to satisfy the investigators. This might potentially introduce social desirability bias or even recollection bias into the study findings. However, the potential bias was mitigated by ensuring that the research participants were guaranteed anonymity and were informed about the significance of providing honest responses.

### Statistical analysis

The data was subjected to analysis using SPSS software, version 25. Descriptive statistics were conducted on all variables included in the study. Continuous data were expressed using means ± standard deviation (SD), whereas categorical variables were expressed using frequencies and percentages. The independent t-test was used to evaluate the differences in the means of continuous variables between men and women participants. The chi-square test was used to evaluate the discrepancy in the categorical variables, namely knowledge and practice, with respect to the genders of the participants. A P-value that falls below the predetermined threshold of 0.05 is considered to possess statistical significance. Moreover, the final study analysis omitted the involvement of 48 people who were part of the pilot phase.

## Results

The study recruited 591 participants. The majority of participants were men (64.8%) with average age of 33.5±9.1 years. More than two-thirds (72.9%) were married and 88.7% had university education. Approximately 65% had governmental work with income between 0.5-1.0 million Iraqi Dinars. Although 59.2% had healthcare worker relatives (HCW), 62.9% of them have not received information about how to dispose unused medications ( Table 1).

**Table 1.** The descriptive statistics of the participants.

Parameter		Subcategories	N	%
Gender		Men	383	64.8
		Women	208	35.2
Marital status		Single	148	25.0
		Married	431	72.9
		Divorced	9	1.5
		Widow	3	0.5
Education		Primary school	12	2.1
		Secondary school	55	9.3
		University	524	88.7
Job		Governmental employee	385	65.1
		Student	69	11.7
		Private sector	58	9.8
		Housewife	36	6.1
		Jobless	32	5.4
		Retired	11	1.9
Monthly income (million Iraqi Dinars)		˂0.5	156	26.4
		0.5-1.0	290	49.1
		˃1.0	145	24.5
Has healthcare worker relative		Yes	350	59.2
		No	241	40.8
Have you received information about how to dispose unused medications		Yes	219	37.1
		No	372	62.9
Age (years)	Minimum	Maximum		
	18.0	70.0	33.45	9.10
Number of family members	1.0	38.0	5.64	3.42

**Figure 1.**
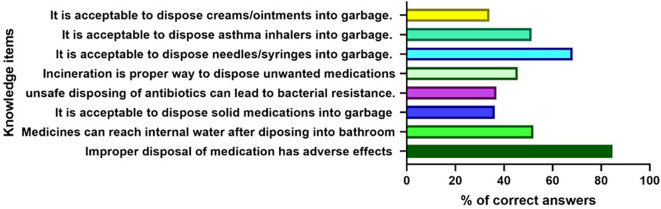
The percent of the correct answers for each knowledge item among the participants.

The majority of participants (˃50%) answered four out of eight knowledge questions correctly. More than 84% correctly agreed that improper disposal of unused and expired medication has adverse effects on the environment. In contrast, only one-third (34-36%) answered correctly that the other three items related to the ways of proper/safe disposal of unused medications. In other words, more than two-thirds of the participants believed that disposal of solid and semi-solid medications into garbage is acceptable. In contrast, 68.2% believed that it is not acceptable to dispose needles and syringes into garbage ( Table 2 and Figure 1).

**Table 2.** The frequency of correct and incorrect answers for the knowledge questions.

Knowledge items	Yes, N (%)	No, N (%)	I do not know, N (%)
Improper disposal of unused and expired medication has adverse effects on environment.	500 (84.6)	12 (2.0)	79 (13.4)
The medications can reach to internal water when they are disposed into bathroom or sink.	308 (52.1)	81 (13.7)	202 (34.2)
It is acceptable to dispose solid medications such as tablets and capsules into garbage (trash) can.	295 (49.9)	214 (36.2)	82 (13.9)
Improper/unsafe disposing of antibiotics such as amoxicillin can lead bacterial resistance.	218 (36.9)	88 (14.9)	285 (48.2)
Incineration is proper environmental way to dispose unwanted medications.	270 (45.7)	214 (36.2)	107 (18.1)
It is acceptable to dispose needles and syringes into garbage.	158 (26.7)	403 (68.2)	30 (5.1)
It is acceptable to dispose asthma inhalers into garbage **.**	200 (33.8)	303 (51.3)	88 (14.9)
It is acceptable to dispose creams and ointments into garbage.	314 (53.1)	201 (34)	76 (12.9)

The majority of participants (60-95%) had positive attitudes toward the necessity of proper disposal of unused medications and the pivotal roles of healthcare workers (HCWs) to raise awareness about the safe disposal methods. The majority of participants (60-95%) agreed with two items describing the HCW roles in counseling about improper ways of disposal. The attitude items indicated two suggested methods to enhance the safe disposal of unused medications including via community pharmacies and encouraging patients to return unused/expired medications to community pharmacies to be disposed safely ( Table 3).

**Table 3.** The attitude of the participants toward the disposal of unused/expired medications.

Attitude items	Strongly disagree, N (%)	Disagree, N (%)	Neutral, N (%)	Agree, N (%)	Strongly agree, N (%)	Mean	ST dev
My responsibility is to protect my family from the adverse effects of unused medications.	1.0 (0.2)	7 (1.2)	45 (7.6)	119 (20.1)	419 (70.9)	4.60	0.70
I am willing to donate my unused medications before their expiration.	6.0 (1)	21 (3.6)	62 (10.5)	207 (35)	295 (49.9)	4.29	0.87
If there is monetary incentive for customers to return unused medicines, I would be more likely to do so.	7.0 (1.2)	40 (6.8)	98 (16.6)	232 (39.3)	214 (36.2)	4.03	0.95
Physicians and pharmacists should provide advice on the safe disposal of unused and expired household medicines.	4.0 (0.7)	3.0 (0.5)	20 (3.4)	134 (22.7)	430 (72.8)	4.66	0.64
Awareness programs about how to dispose of unused and expired medicines should be initiated.	1.0 (0.2)	5.0 (0.8)	16 (2.7)	146 (24.7)	423 (71.6)	4.67	0.59
There should be a public awareness program about the harmful effects of improper medicine disposal practices.	3.0 (0.5)	6.0 (1.0)	14 (2.4)	162 (27.4)	406 (68.7)	4.63	0.64
Community pharmacists have an important role in mitigating the problem of improper medicine disposal practices.	28 (4.7)	53 (9.0)	133 (22.5)	170 (28.8)	207 (35)	3.80	1.15
There is lack of adequate information on the safe disposal of unused and expired medications.	3.0 (0.5)	8.0 (1.4)	83 (14)	212 (35.9)	285 (48.2)	4.30	0.80
Children are at high risk of unused and expired medications.	2.0 (0.3)	7.0 (1.2)	51 (8.6)	167 (28.3)	364 (61.6)	4.50	0.73
Returning of unused or expired medications to the pharmacy for disposal would be convenient.	13 (2.2)	75 (12.7)	138 (23.4)	200 (33.8)	165 (27.9)	3.73	1.07

Although the majority of the participants had a positive attitude toward the safe disposal of unused/expired medications, their actual practice is improper disposal for these medications ( Table 4). Approximately 70% of the participants have disposed unused/expired medications into their home trash can. Additionally, 66.3% of them had unused medications in their homes ( Table 4).

**Table 4.** The practice of the participants toward the disposal of unused/expired medications.

Practice items	Subcategories	N	%
Do you have unused medications in your home?	Yes	392	66.3
	No	199	33.7
I dispose my medications when they change their color or taste.	Yes	494	83.6
	No	97	16.4
How often do you check your medications’ expiration date?	Never	26	4.4
	Rarely	50	8.5
	Sometimes	74	12.5
	Often	116	19.6
	Always	325	55.0
How do you dispose your expired medications?	Flush down into toilet or sink	25	4.2
	Home trash can	415	70.2
	Incineration	81	13.7
	Others	70	11.8
Other methods of medication disposing.	landfill	31	5.24
	Burning	10	1.69
	Deep burial	7	1.18
	Return to pharmacy	8	1.35
	Give it to friends	4	0.7
	I don’t know	10	1.69

According to the participants’ gender, there was significant (P-value˂0.05) differences in seven out of eight knowledge items related to disposing of unused/expired medications. In other words, men participants had significantly higher percentages of correct answers in almost all knowledge items compared to women participants ( Table 5).

**Table 5.** The difference in the participants’ knowledge about medication disposal according to the gender.

Knowledge items			Gender		P-value
			Men	Women	
Improper disposal of unused and expired medication has adverse effects on environment.	Correct	Count	341	159	0.000 *
		%	89.0%	76.4%	
	Incorrect	Count	42	49	
		%	11.0%	23.6%	
The medications can reach to internal water when they are disposed into bathroom or sink.	Correct	Count	213	95	0.021 *
		%	55.6%	45.7%	
	Incorrect	Count	170	113	
		%	44.4%	54.3%	
It is acceptable to dispose solid medications such as tablets and capsules into garbage (trash) can.	Correct	Count	168	46	0.000 *
		%	43.9%	22.1%	
	Incorrect	Count	215	162	
		%	56.1%	77.9%	
Improper/unsafe disposing of antibiotics such as amoxicillin can lead bacterial resistance.	Correct	Count	150	68	0.119
		%	39.2%	32.7%	
	Incorrect	Count	233	140	
		%	60.8%	67.3%	
Incineration is proper environmental way to dispose unwanted medications.	Correct	Count	208	62	0.000 *
		%	54.3%	29.8%	
	Incorrect	Count	175	146	
		%	45.7%	70.2%	
It is acceptable to dispose needles and syringes into garbage.	Correct	Count	272	131	0.045 *
		%	71.0%	63.0%	
	Incorrect	Count	111	77	
		%	29.0%	37.0%	
It is acceptable to dispose asthma inhalers into garbage.	Correct	Count	211	92	0.012 *
		%	55.1%	44.2%	
	Incorrect	Count	172	116	
		%	44.9%	55.8%	
It is acceptable to dispose creams and ointments into garbage.	Correct	Count	153	48	0.000 *
		%	39.9%	23.1%	
	Incorrect	Count	230	160	
		%	60.1%	76.9%	

* Significant (P-value˂0.05) according to Pearson Chi-Square.

There was significant (P-value˂0.05) difference in the participant attitude toward one item regarding the disposal of unused/expired medications according to the gender. In other words, men participants more likely to believe “if there is monetary incentive for customers to return unused medicines, they would be more likely to do so”. However, there was no other significant (P-value˃0.05) difference according to the participant gender with the other nine attitude items ( Table 6).

**Table 6.** The difference in the participants’ attitude about medication disposal according to the gender.

Attitude items	Gender	N	Mean	Std. Deviation	P-value
My responsibility is to protect my family from the adverse effects of unused medications.	Men	383	4.63	0.66	0.149
	Women	208	4.55	0.75	
I am willing to donate my unused medications before their expiration.	Men	383	4.28	0.86	0.758
	Women	208	4.31	0.88	
If there is monetary incentive for customers to return unused medicines, I would be more likely to do so.	Men	383	4.09	0.89	0.045 *
	Women	208	3.91	1.05	
Physicians and pharmacists should provide advice on the safe disposal of unused and expired household medicines.	Men	383	4.63	0.65	0.094
	Women	208	4.72	0.60	
Awareness programs about how to dispose of unused and expired medicines should be initiated.	Men	383	4.64	0.61	0.131
	Women	208	4.72	0.54	
There should be a public awareness program about the harmful effects of improper medicine disposal practices.	Men	383	4.60	0.64	0.201
	Women	208	4.67	0.62	
Community pharmacists have an important role in mitigating the problem of improper medicine disposal practices.	Men	383	3.83	1.18	0.493
	Women	208	3.76	1.10	
There is lack of adequate information on the safe disposal of unused and expired medications.	Men	383	4.27	0.80	0.247
	Women	208	4.35	0.78	
Children are at high risk of unused and expired medications.	Men	383	4.50	0.73	0.714
	Women	208	4.48	0.74	
Returning of unused or expired medications to the pharmacy for disposal would be convenient.	Men	383	3.77	1.02	0.198
	Women	208	3.65	1.15	

* Significant (P-value˂0.05) according to Independent T-test.

There were significant (P-value˂0.05) differences in four knowledge items about disposing of unused/expired medications according to the education level as presented in Table 7. In other words, participants with university education had a significantly higher percentage of correct answers to three of the knowledge questions. In contrast, the participants with primary school education had significantly higher percentage of correct answer to one knowledge item: “Incineration is proper environmental way to dispose unwanted medications.

**Table 7.** The association between education level and the knowledge about disposing unused/expired medications.

Knowledge items			Education level			
			Primary	Secondary	University	P-value
‡ Improper disposal of unused and expired medication has adverse effects on environment.	Correct	Count	10	42	448	0.164
		%	83.3%	76.4%	85.5%	
	Incorrect	Count	2	13	76	
		%	16.7%	23.6%	14.5%	
The medications can reach to internal water when they are disposed into bathroom or sink.	Correct	Count	4	26	278	0.302
		%	33.3%	47.3%	53.1%	
	Incorrect	Count	8	29	246	
		%	66.7%	52.7%	46.9%	
‡ It is acceptable to dispose solid medications such as tablets and capsules into garbage (trash) can.	Correct	Count	2	7	205	0.000 *
		%	16.7%	12.7%	39.1%	
	Incorrect	Count	10	48	319	
		%	83.3%	87.3%	60.9%	
‡ Improper/unsafe disposing of antibiotics such as amoxicillin can lead bacterial resistance.	Correct	Count	4	17	197	0.643
		%	33.3%	30.9%	37.6%	
	Incorrect	Count	8	38	327	
		%	66.7%	69.1%	62.4%	
Incineration is proper environmental way to dispose unwanted medications.	Correct	Count	11	28	231	0.003 *
		%	91.7%	50.9%	44.1%	
	Incorrect	Count	1	27	293	
		%	8.3%	49.1%	55.9%	
‡ It is acceptable to dispose needles and syringes into garbage.	Correct	Count	5	27	371	0.001 *
		%	41.7%	49.1%	70.8%	
	Incorrect	Count	7	28	153	
		%	58.3%	50.9%	29.2%	
It is acceptable to dispose asthma inhalers into garbage.	Correct	Count	4	19	280	0.012 *
		%	33.3%	34.5%	53.4%	
	Incorrect	Count	8	36	244	
		%	66.7%	65.5%	46.6%	
It is acceptable to dispose creams and ointments into garbage.	Correct	Count	5	11	185	0.053
		%	41.7%	20.0%	35.3%	
	Incorrect	Count	7	44	339	
		%	58.3%	80.0%	64.7%	

* Significant (P-value˂0.05) according to Chi-square or.

^‡^ Fisher's Exact Test.

There were significant (P-value˂0.05) differences in three knowledge items about disposing of unused/expired medications according to participant age. In other words, older participants had significantly higher knowledge about the disposing of unused/expired medications compared to younger ones ( Table 8).

**Table 8.** The difference in the participants’ knowledge about disposing unused/expired medications according to their age.

Knowledge items			Age (years)		
	Answers	N	Mean	Std. Deviation	P-value
Improper disposal of unused and expired medication has adverse effects on environment.	Correct	500	33.84	9.31	0.015 *
	Incorrect	91	31.32	7.53	
The medications can reach to internal water when they are disposed into bathroom or sink.	Correct	308	33.45	9.79	0.993
	Incorrect	283	33.45	8.30	
It is acceptable to dispose solid medications such as tablets and capsules into garbage (trash) can.	Correct	214	33.97	8.65	0.292
	Incorrect	377	33.15	9.34	
Improper/unsafe disposing of antibiotics such as amoxicillin can lead bacterial resistance.	Correct	218	33.39	8.37	0.898
	Incorrect	373	33.49	9.51	
Incineration is proper environmental way to dispose unwanted medications.	Correct	270	34.59	9.56	0.005 *
	Incorrect	321	32.49	8.58	
It is acceptable to dispose needles and syringes into garbage.	Correct	403	33.39	8.87	0.818
	Incorrect	188	33.57	9.59	
It is acceptable to dispose asthma inhalers into garbage.	Correct	303	33.65	8.88	0.581
	Incorrect	288	33.24	9.33	
It is acceptable to dispose creams and ointments into garbage.	Correct	201	34.48	9.86	0.047 *
	Incorrect	390	32.92	8.65	

* Significant (P-value˂0.05) according to Independent T-test.

According to the Chi-square test, the participants with higher income were more likely to have correct answers regarding four knowledge items compared to lower income participants ( Table 9). In other words, higher income was associated with better knowledge about disposing unused/expired medications.

**Table 9.** The association between the participant income levels and their knowledge about disposing unused/expired medications.

Knowledge items			Monthly income (Million Iraqi Dinars)			P-value
			˂0.5	0.5-1.0	˃1.0	
Improper disposal of unused and expired medication has adverse effects on environment.	Correct	Count	119	251	130	0.002 *
		%	76.3%	86.6%	89.7%	
	Incorrect	Count	37	39	15	
		%	23.7%	13.4%	10.3%	
The medications can reach to internal water when they are disposed into bathroom or sink.	Correct	Count	71	150	87	0.041 *
		%	45.5%	51.7%	60.0%	
	Incorrect	Count	85	140	58	
		%	54.5%	48.3%	40.0%	
It is acceptable to dispose solid medications such as tablets and capsules into garbage (trash) can.	Correct	Count	41	103	70	0.000 *
		%	26.3%	35.5%	48.3%	
	Incorrect	Count	115	187	75	
		%	73.7%	64.5%	51.7%	
Improper disposing of antibiotics such as amoxicillin can lead bacterial resistance.	Correct	Count	50	105	63	0.120
		%	32.1%	36.2%	43.4%	
	Incorrect	Count	106	185	82	
		%	67.9%	63.8%	56.6%	
Incineration is proper environmental way to dispose unwanted medications.	Correct	Count	69	135	66	0.897
		%	44.2%	46.6%	45.5%	
	Incorrect	Count	87	155	79	
		%	55.8%	53.4%	54.5%	
It is acceptable to dispose needles and syringes into garbage.	Correct	Count	94	200	109	0.019 *
		%	60.3%	69.0%	75.2%	
	Incorrect	Count	62	90	36	
		%	39.7%	31.0%	24.8%	
It is acceptable to dispose asthma inhalers into garbage.	Correct	Count	72	153	78	0.320
		%	46.2%	52.8%	53.8%	
	Incorrect	Count	84	137	67	
		%	53.8%	47.2%	46.2%	
It is acceptable to dispose creams and ointments into garbage.	Correct	Count	55	87	59	0.079
		%	35.3%	30.0%	40.7%	
	Incorrect	Count	101	203	86	
		%	64.7%	70.0%	59.3%	

* Significant (P-value˂0.05) according to Chi-square test.

## Discussion

The increasing availability and use of medicines have resulted in accumulated and inadequate disposal of unwanted, expired drugs inside households, which may give rise to environmental and public health concerns. ^32^^,^^33^ The reduction of this issue may be achieved by the implementation of policies and recommendations for medication disposal, ^34^ as well as by enhancing public knowledge about suitable techniques and procedures for disposing of medicine. ^35^ Hence, the primary objective of this research was to evaluate the general population’s knowledge, attitude, and behavior pertaining to the disposal of unused and expired home medications.

The findings of our study revealed a satisfactory level of knowledge among the general populace about the proper disposal of medications. A majority of the participants correctly answered four out of eight knowledge-based questions related to medicine disposal. Additionally, most of the respondents expressed the belief that it is unacceptable to dispose of needles and syringes in regular rubbish. The present results are consistent with the earlier findings published by A. Althagafi *et al.* in Saudi Arabi, which recruited 1100 participants, ^36^ however they differ with the findings of research done in Lebanon, which emphasized the limited understanding of the general community about medicine disposal, and where the knowledge score were 24.5%. ^6^

Previous research has shown that individuals with intermediate and higher education levels (secondary, university, or postgraduate) and higher incomes had a higher perceived knowledge score, as documented in previous study. ^37^ One reasonable assumption is that those who have higher levels of education and have more financial stability are inclined to exhibit a more intuitive preference towards seeking knowledge and possess a heightened understanding of their environment. In contrast, in line with findings from previous studies, those with lower levels of education are more prone to encountering challenges when attempting to acquire new knowledge and may lack awareness of the potential repercussions of incorrectly disposing of medications. ^38^ The aforementioned findings are consistent with the current study results, indicating that those with a university degree exhibited a statistically significant increase in the proportion of accurate responses to three of the knowledge-based questions.

There was a substantial association seen between gender and knowledge and attitudes, with men exhibiting considerably higher scores in both domains. The results exhibit inconsistency compared to prior research, which indicated that women showed a considerably higher inclination towards using medicine collecting facilities than men in a study conducted between Lebanese and Malaysian populations. ^6^^,^^39^ Additionally, women were found to possess a greater sense of personal accountability towards the proper disposal of medication. ^40^

The respondents expressed a belief in the need of consulting healthcare experts on appropriate means of disposal. This finding is consistent with the expectation that health care providers bear primary responsibility for managing medicines and hazardous waste. ^41^

The present study reveals that 66.3% of the participants had leftover, unutilized, or unwanted medicines, a finding consistent with previous research done in India and Harar city in Ethiopia, where 68% and 66% of the respondents, respectively, retained unused medicines inside their households. ^17^^,^^42^ Furthermore, the findings of research done in Northern Ethiopia by Halefom Kahsay *et al*. demonstrated a lesser value compared to the information presented in this study, approximately 52.4% of the participants said they had leftover medications stashed inside their households, with analgesics being the most prevalent category, accounting for 41.5% of the reported instances. ^43^

The results from the study indicate that a significant proportion of respondents reported disposing of expired drugs in their home garbage (approximately 70%), in line with previous research that gave insight regarding a number of studies undertaken in different countries around the world. ^18^ In Busan city, Korea, housewives were seen to dispose of unneeded drugs by using regular rubbish bags. ^44^ It was believed that the appropriate approach for disposing of unneeded or expired prescriptions was to flush them down the toilet or drain rather than putting them in the garbage. This was based on the concern that if medications were thrown in the trash, there was a higher likelihood of animals or people coming into contact with them. ^45^ A small proportion, about 4.2% of the survey participants, reported disposing of expired prescriptions by flushing them down the toilet or sink. This disposal method aligns with the patterns seen in Kuwait (2%), and much lower than what reported previously in the United States, as it was documented that 35.4% of participants flushed medications down the toilet or sink. ^21^^,^^46^ Notably, this approach is considered the recommended practice for liquid pharmaceuticals. ^18^ A small portion of the participants in the study engaged in the practice of returning drugs that were unused and had expired to drugstores, but only 1.35%. This behavior has similarities to comparable practices seen in the United States and Malaysia as reported in previous studies. ^46^^,^^47^ The various methods used for the safe disposal of residual pharmaceuticals have a substantial impact on mitigating the entry of these substances into the environment since they have the potential to pose risks to both the environment and individuals. ^48^^,^^49^

### Limitations

It is crucial to recognize that this research has experienced several limitations. The results were contingent upon the accuracy and sincerity of the participants’ responses, which may introduce a bias in the information obtained. There was no physical inspection conducted on the storage of medication. Moreover, while the existing sample size might consider enough for achieving statistical significance, it is recommended to use a bigger sample size to improve the applicability of the findings to the wider community. The last limitation of the research design is its cross-sectional character, which hinders our ability to establish causal relationships between the selected factors and outcome variables across time.

## Conclusion

The present study conducted among the general population in Iraq provides insights into the actual practices and level of knowledge about the appropriate handling and disposal methods of unused and expired medications. Despite the widespread awareness among the general community of the potential damage and significant risks associated with expired pharmaceuticals, a lack of knowledge was observed on the appropriate methods for disposal and management of such medications. A significant proportion of the participants had positive attitudes about the necessity of appropriately disposing of unneeded drugs. Yet, their current practice involves poor disposal methods for these drugs. The healthcare system and its practitioners must play a pivotal role in providing education and guidance to the general population in this matter. It is recommended that authorities take proactive measures to develop and implement medication take-back campaigns, along with methods aimed at increasing public awareness on this issue. Lastly, all community members must take up the duty and responsibility of protecting the environment and preventing the unethical discharge of chemical substances, such as drugs, that may harm the natural environment.

### Implications for practice

Our findings underscore the need to implement a comprehensive waste management plan at the national level. This strategy should clearly outline the responsibilities of healthcare practitioners, governmental and non-governmental entities, and the pharmaceutical sector. Various stakeholders, such as broadcasting stations, educational institutions, pharmacists, and healthcare practitioners, may contribute to disseminating knowledge via educational initiatives. These efforts aim to enhance public understanding of the environmental implications associated with pharmaceutical residues and promote appropriate practices and disposal standards to mitigate medicine waste.

## Data Availability

Zenodo: Assessment of general population knowledge, attitude, and practice on safe unused and expired drugs disposal: a cross-sectional study, https://doi.org/10.5281/zenodo.8407783. ^50^ This project contains the following underlying data:
- Underlying data new version.xlsx Data are available under the terms of the Creative Commons Attribution 4.0 International license (CC-BY 4.0). Zenodo: Assessment of general population knowledge, attitude, and practice on safe unused and expired drugs disposal: a cross-sectional study [questionnaire], https://doi.org/10.5281/zenodo.8408349. ^51^ This project contains the following extended data:
- General population questionnaire.pdf Data are available under the terms of the Creative Commons Attribution 4.0 International license (CC-BY 4.0).
