# Supplementary material for: Assessment of general population knowledge, attitude, and practice on safe unused and expired drugs disposal: a cross-sectional study
Source: F1000Res. 2023 Oct 16;12:1333. [Version 1] doi: 10.12688/f1000research.142146.1 (PMC10955187; doi:10.12688/f1000research.142146.1)
Supplement: Peer review history

# Reviewer report: Reviewer response for version 1

**DOI:** 10.5256/f1000research.155652.r235068

**Published:** 2024-03-20

**Authors:** Miteshkumar Maurya

**Affiliations:** 1 Curative Clinic, Curative Clinic, Mumbai, Maharashtra, India

This cross-sectional study is well designed and executed and gives good information for specified population. 

However, kindly address the following points-

 1. Basis of calculation of sample size need justification/reference eg. based on response rate of previous published study and 

 what response rate could you get out of total targeted sample size

 2. Elaborate some more points on the inclusion and exclusion criteria eg. some participants would not have access to emails and may have not been able to participate in survey adding to bias in the result outcomes. May be your results are reflective of literate/ educated population KAP having access to google forms only. Please justify if you have overcome these challenges/or specify in limitations.

 3. Please add a reference of an article you used for content validity method for your questionnaire. 

 4. You may site this relevant article -Munshi R, Dhiman A, Maurya M. A cross-sectional survey to assess the drug disposal practices of unused and expired medicines among lay public visiting a tertiary care hospital in an urban metropolis. Indian J Physiol Pharmacol 2023;67:29-35.

Is the work clearly and accurately presented and does it cite the current literature?

Partly

If applicable, is the statistical analysis and its interpretation appropriate?

Partly

Are all the source data underlying the results available to ensure full reproducibility?

Yes

Is the study design appropriate and is the work technically sound?

Yes

Are the conclusions drawn adequately supported by the results?

Yes

Are sufficient details of methods and analysis provided to allow replication by others?

Partly

Reviewer Expertise:

Pharmacology and Therapeutics, Clinical Pharmacology, Pharmacogenetics, Pharmacovigilance

I confirm that I have read this submission and believe that I have an appropriate level of expertise to confirm that it is of an acceptable scientific standard, however I have significant reservations, as outlined above.

# Reviewer report: Reviewer response for version 1

**DOI:** 10.5256/f1000research.155652.r235073

**Published:** 2024-03-06

**Authors:** Souraya Domiati

**Affiliations:** 1 Beirut Arab University, Beirut, Beirut Governorate, Lebanon

The article overall is well designed and written yet there are some comments.

 Abstract: 

 1. The background in the abstract should be rewritten especially the first sentence stating that it is" well-recognized issue that has convened growing recognition in several contexts" (recognized and recognition)

 2. 50% is not the majority.

 3. what is meant in the conclusion by prescriptions? Please change the word.

 Method:

 1. Please state the name of the ethical guidelines and regulations you followed.

 2. Did you check for the normality of your data?

 Results:

 1. omit "that" in the second paragraph.

 2. No need for figure 1. It is just a repetition of the table 2.

 3. Please specify, in the method section, how the attitude was scored using the 5 point Likert scale. Is neutral calculated as 3??

 4. Please double-check if your data was normally distributed otherwise you have to use non-parametric tests.

 5. The knowledge score was not shown in the results.

 Discussion:

 1. If the participants answered 4 items out of 8 correctly, we cannot conclude that their knowledge is satisfactory. 

 2. You assumed that " those who have higher levels of education and have more financial stability are inclined to exhibit a more intuitive preference towards seeking knowledge" but you did not do a multivariate analysis. You just analyzed every item alone.

Is the work clearly and accurately presented and does it cite the current literature?

Yes

If applicable, is the statistical analysis and its interpretation appropriate?

Partly

Are all the source data underlying the results available to ensure full reproducibility?

Yes

Is the study design appropriate and is the work technically sound?

Yes

Are the conclusions drawn adequately supported by the results?

Yes

Are sufficient details of methods and analysis provided to allow replication by others?

Yes

Reviewer Expertise:

Pharmacology and epidemiology

I confirm that I have read this submission and believe that I have an appropriate level of expertise to confirm that it is of an acceptable scientific standard, however I have significant reservations, as outlined above.

# Reviewer report: Reviewer response for version 1

**DOI:** 10.5256/f1000research.155652.r235077

**Published:** 2024-02-05

**Authors:** Sangeetha Raja

**Affiliations:** 1 SRM Medical College Hospital and Research Centre, Chennai, Tamil Nadu, India

The paper exhibits overall clarity in language, though some sentences could be refined for enhanced coherence. While the paper's structure is well-organized, smoother transitions between sections would improve the reading experience. The introduction effectively emphasizes global concerns about medication disposal but lacks specificity regarding the issue's prevalence in Iraq, diminishing its regional impact. Strengthening this with specific information on the region would enhance the introduction.

 The study design and data collection methods are well-described, yet additional details on questionnaire development, validation, and the pilot phase would augment methodological rigor. The mention of ethical approval is present, but further elaboration on ethical considerations, particularly the voluntary nature of participation and anonymity assurance, would be beneficial. The rationale for the sample size of 591 individuals needs more justification, and clarifying the exclusion of healthcare workers is advisable.

 The comprehensive results section covers participant demographics, knowledge, attitudes, and practices. Presenting data visually, such as through tables and figures, would enhance readability. The discussion adeptly compares findings with existing literature but lacks a deeper analysis of implications for public health and the environment.

 While limitations are appropriately acknowledged, discussing how these limitations might influence study outcomes would enhance transparency. The conclusion succinctly summarizes key findings, but reinforcing practical implications and offering specific recommendations for policymakers, healthcare providers, and the general public based on study findings would strengthen this section.

Is the work clearly and accurately presented and does it cite the current literature?

Partly

If applicable, is the statistical analysis and its interpretation appropriate?

I cannot comment. A qualified statistician is required.

Are all the source data underlying the results available to ensure full reproducibility?

No source data required

Is the study design appropriate and is the work technically sound?

Yes

Are the conclusions drawn adequately supported by the results?

Yes

Are sufficient details of methods and analysis provided to allow replication by others?

Yes

Reviewer Expertise:

Pharmacogenomics, Preclinical studies ,environmental study

I confirm that I have read this submission and believe that I have an appropriate level of expertise to confirm that it is of an acceptable scientific standard.
